# Using Long Short-Term Memory for Building Outdoor Agricultural Machinery

**DOI:** 10.3389/fnbot.2020.00027

**Published:** 2020-05-29

**Authors:** Chien-Hung Wu, Chun-Yi Lu, Jun-We Zhan, Hsin-Te Wu

**Affiliations:** ^1^Department of Marine Recreation, National Penghu University of Science and Technology, Magong, Taiwan; ^2^Department of Information Management, National Penghu University of Science and Technology, Magong, Taiwan; ^3^Department of Computer Science and Information Engineering, National Penghu University of Science and Technology, Magong, Taiwan; ^4^Department of Computer Science and Information Engineering, National Ilan University, Yilan City, Taiwan

**Keywords:** artificial intelligence, robot, deep learning, intelligent agriculture, automation equipment, long short-term memory

## Abstract

Today, climate change has caused a decrease in agricultural output or overall yields that are not as expected; however, with the ongoing population explosion, many undeveloped countries have transformed into emerging countries and have transformed farmland to be used in other types of applications. The resulting decline in agricultural output further increases the severity of the food crisis. In this context, this study proposes an outdoor agricultural robot that uses Long Short-Term Memory (LSTM). The key features of this innovation include: (1) the robot is portable, and it uses green power to reduce installation cost, (2) the system combines the current environment with weather forecasts through LSTM to predict the correct timing for watering, (3) detecting the environment and utilizing information from weather forecasts can help the system to ensure that growing conditions are suitable for the crops, and (4) the robot is mainly for outdoor applications because such farms lack sufficient electricity and water resources, which makes the robot critical for environmental control and resource allocation. The experimental results indicate that the robot developed in this study can detect the environment effectively to control electricity and water resources. Additionally, because the system is planned to increase agricultural output significantly, the study predicts the variables through multivariate LSTM, which controls the power supply from the solar power system.

## 1. Introduction

The world is currently facing energy and food crises; moreover, many countries are transforming farmland into industrial land for related usage because they are transforming from being undeveloped to being emerging countries. All of these factors have reduced agricultural output tremendously. The literature (Liu et al., 2018) has mentioned that rapid economic development and urbanization are consuming the resources of the planet extremely rapidly and that, especially due to the impact of climate change, the yields of many crops are declining or are not as expected. Therefore, the huge challenge arises of rethinking our exploitation of food and energy. On the other hand, traditional agriculture requires labor and machinery to irrigate and harvest; yet, with the impact of an aging agricultural labor population, a large amount of farmland is deserted or fallow. Consequently, agriculture needs to be upgraded to intelligent production, which could increase output while reducing labor because farmers would then only need to calibrate the smart equipment and set relevant parameters.

Precision Agriculture (PA) works to detect relevant environmental information around the farmland to enhance automated production; the PA system controls automated machinery and the related irrigation equipment while farmers will only need to calibrate the equipment and confirm correctness; this will reduce agricultural labor and ensure a good crop-growing environment Narvaez et al. (2017). Hu et al. (2019) utilized an intelligent agriculture system to handle the tasks of irrigation and production, as well as doing the watering work according to the environmental conditions of the farmland to manage the growing environment. In another study Ayaz et al. (2019), the farmland environment was detected via the Internet of Things (IoT), which supports equipment, such as lighting and watering equipment, to make sure that the crops grow in a suitable environment. Furthermore, the article pointed out that for farmland in extreme climate areas, there are difficulties over a huge amount of farmland in planting proper crops to grow in such an environment; thus, the development of smart agriculture can re-analyze farmland environments to find suitable species of crops for the farmers. Finally, a study by Chebrolu et al. (2018) used unmanned aerial vehicles to check and monitor farms; these can detect the environment and ease the agricultural labor issue.

Based on the aforementioned issues, this study offers an approach for building outdoor agricultural machinery that utilizes LSTM. Additionally, because most farmland is located beyond the reach of power equipment or where there are not enough water resources, this study proposes a system that uses solar power as the key electricity supply and makes the equipment movable. Due to the limited electricity offered by the solar power system, the approach detects the equipment and conducts watering tasks by LSTM to avoid energy waste, the detectors used in the experiment monitor the soil temperature and humidity, pH value, and sunlight conditions in the farmland. The study also collects forecast information, such as temperature and the Probability of Precipitation, from the Central Weather Bureau and uses this to predict soil humidity and sunlight conditions via LSTM. The robot will forecast the best time to activate the watering equipment, and when the prediction result exceeds the set suitable conditions, the server will notify the farmer. This approach is an application designed for outdoor usage and is shown to be practical on a farm. The experimental result proves that the proposed method is feasible; moreover, the equipment presented is cost-efficient, which makes it well-suited to widespread distribution and massive adoption for general applications.

## 2. Related Work

To date, many studies have offered intelligent agriculture-based approaches for improving the quality of crops. A study by Liu et al. (2018) mainly used natural resources to support farm planting; for example, it used overproduced energy to provide watering and lighting or utilized solar energy and rainwater to take care of the farm. Using natural resources can significantly reduce resource waste and achieve eco-friendliness. Narvaez et al. (2017) used spectral data to monitor the growing conditions of crops and decide whether to trim or spray pesticides. Hu et al. (2019) utilized wireless sensor networks to transfer detector data and reduce installation cost as well as using the network to lower the energy consumption of the detectors. Ayaz et al. (2019), meanwhile, detailed the structures of intelligent agriculture performed through IoT and cloud networks. IoT equipment supports the delivery of the environmental data collected to a cloud server, which enables agricultural experts to analyze data for decision-making. Two other studies, Chebrolu et al. (2018) and Farooq et al. (2019) utilized unmanned aerial vehicles to monitor farmland regularly to detect the growing conditions of the crops; additionally, Shadrin et al. (2019) used a Convolutional Neural Network to judge whether the growing conditions had reached expectations, and Bayrakdar (2019) combined detectors with wireless sensor networks to check whether there were holes underneath the farmland and used sonar to determine whether the area was suffering from plant diseases and pest damage. The method presented in this article can detect the growing environment of the crop regularly to ensure the quality of the crop.

Because Internet equipment cannot be robustly installed in outdoor farms, it is better to develop a Wi-Fi system to transmit the data and ensure delivery quality. A study by Chen and Yang (2019) offers an intelligent agriculture method to improve growing for farmers significantly by using relevant data to build decision systems or Knowledge-Based Systems. Lozoya et al. (2016) constructed modular agriculture that helps diverse farms to adjust system modules based on their requirements, which ensures an optimal automated process. Herrera et al. (2016) combine four-wheeled vehicles with sensors to tour around the farm; with fixed routes, the vehicle would collect the growth information of each selected part and transfer the data back for analysis. The method presented by Murugan et al. (2017) can be used in latifundios (large farms) because it uses aerial shots to identify crop maturity; this method can substantially reduce agricultural labor pressures. Another study, Elijah et al. (2018) developed a wireless sensor network system for farmland that can collect farm information effectively; with data analytics, the system could activate the equipment rapidly and ensure that crops were growing under the most suitable environment. In Liu et al. (2019) study combining IoT and cloud computing to record the relevant environmental factors of the farm, the crop quality was increased because the system protected the farm from pollution. Lin et al. (2020) used R-CNN to conduct pest control and detection; instead, this study suggests using a 4G network to improve transmission quality.

Palangi et al. (2016) searched keywords online through LSTM; due to the massive amount of data on the Internet, LSTM helps find the required files rapidly by utilizing the concept of keywords. Wang et al. (2018) examined the production yield of wafers via bilateral LSTM. Park et al. (2018) mainly used LSTM to develop keyword recognition in speech recognition for drivers; because the level of device computation available in a car is usually low, keywords are required to boost searches to fulfill the needs of drivers. Zhou et al. (2019) presented a system that predicts sunlight and estimates the electricity produced by solar power systems through LSTM, which becomes the basis of solar power equipment management. Finally, Zhang et al. (2019) utilized LSTM in edge computing; when video files are too large, an LSTM technique helps predict the buffer memory for each edge computing, node. Guardo et al. (2018) mainly used Fog Computing to reduce the workload of centralized servers. Additionally, Arachchi et al. (2019) scheduled video time arrangements via LSTM, which is beneficial for composing various videos chronologically. The current article uses the LSTM technique to detect the experimental environment and effectively control water resources and electrical power conditions.

In this study, outdoor agricultural machinery was built that has a solar power system to provide electricity because outdoor farms usually lack sufficient electricity and water resources. Due to limited solar power energy, for achieving the performance of monitoring the farm environment and maintaining good growing conditions, the experiment used LSTM to monitor the environment and control the watering system. It combined information on sunlight, soil humidity, temperature, and weather forecasts from the Central Weather Bureau with LSTM to set system schedules and avoid wastage of electricity and water resources.

## 3. The Proposed Scheme

This chapter introduces the following details: the system model; signal delivery and control; data normalization; long short-term memory prediction.

### 3.1. System Model

**Figure 1:**
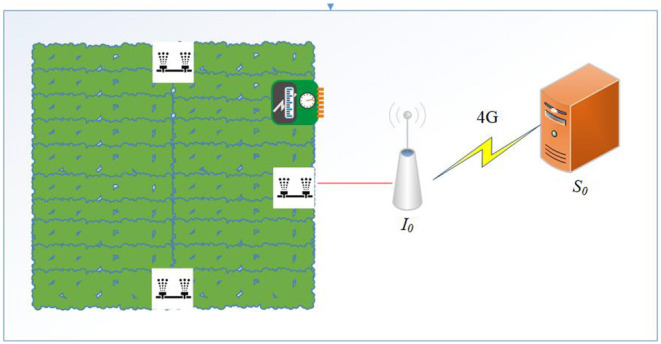
System model.

The article proposes the system model shown in Figure 1. The equipment includes lighting and sensors that detect the barometric pressure, soil temperature and humidity, and pH value in an outdoor farm, and also a sprinkler motor. We attached an IoT development board to the machinery (*I*_0_) that uses stored electricity from the solar power system; the sensors on the equipment deliver the collected data to the server (*S*_0_) via 4G networks. Furthermore, the server updates the weather forecast data through a web crawler and combines this with the sensor data to conduct LSTM analysis. The LSTM system will set watering schedules based on the analytic results to ensure that the growing environment provides a suitable humidity and temperature for the crops. On the other hand, outdoor farmland usually lacks sufficient water resources and electricity; in particular, the installation cost of electrical equipment is a huge burden for farmers. Therefore, this research presents a solar power system for reducing the cost stress; additionally, the designed equipment is movable to any location on the farm, which also reduces the workload of farmers. The system uses LSTM to predict soil conditions and weather changes, the equipment sets the schedules for activating the machinery, and the design saves electricity to avoid waste; all of these improve the growing environment for crops.

The research developed an outdoor robot for detecting environmental conditions and controlling the watering system. The robot transmits the data to the server for further analysis, and the server can use environmental factors and weather forecasts to predict the environmental conditions. Different kinds of crops grow under different conditions; hence, through the analysis, the result can help to judge whether the environmental conditions are beyond expectations. If the growing conditions are not suitable for growing the crop, the system will warn the farmers. Moreover, because weather data exhibits linear growth, the study applies LSTM to calculate environmental variables so as to predict the environmental factors and conditions of the farmland to increase output.

### 3.2. Signal Delivery and Control

**Figure 2:**
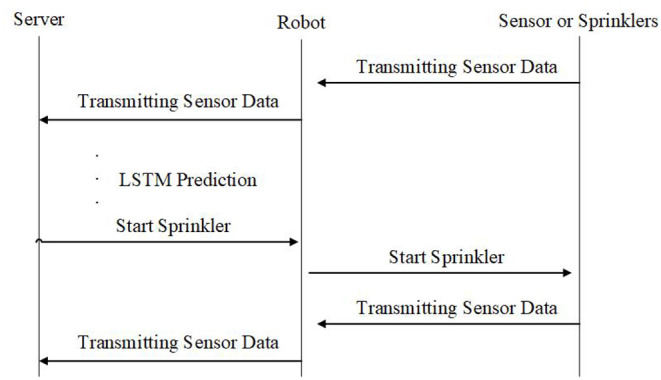
Signal delivery and control model.

This study uses Extensible Messaging and Presence Protocol (XMPP) to collect information from the sensors on the machine and uses LSTM to predict soil temperature and humidity so as to judge the activation time of the equipment. As shown in Figure 2, the machinery and the IoT development board will maintain a low power consumption status. When the LSTM system suggests starting the equipment, the server will send a signal to the IoT development board to activate the sprinkler system and the sensor. When the task is finished, the IoT development board will also send a signal back to the server and provide the sensor data for analysis. Due to the low power consumption, the IoT development board can avoid wastage of electricity; hence, when the server receives the sensor data, it will further conduct LSTM prediction analysis and set the next schedule for activation.

### 3.3. Data Normalization

The machinery with sensors can detect soil temperature and humidity, sunlight, and related information, which will be delivered to the server through 4G networks. Nonetheless, packet loss might occasionally occur, causing data error, and insects might also cause the sensors to detect incorrect information; thus, it is necessary to judge the data correctness. Firstly, to check the correctness of the detected temperature, we input the lowest and highest temperatures from the Central Weather Bureau and check the current time to judge the rationality. The algorithm is shown below:

**Algorithm 1:** Temperature detection algorithm.

//If the value of the current temperature minus the estimated temperature is smaller than a set threshold.
**if** (|*Te*_*ct*_ − *PTe*_*s*_|) < *Threshold* **then**
Correct
**else**
Re-detect
**end if**

*Te*_*ct*_ means the current soil temperature detected from the sensor, and *PTe*_*s*_ is the temperature loaded from the Central Weather Bureau. If the temperature between 06:00 and 17:00 is set to be the highest temperature, the temperature during the rest of the day will be set to be the lowest temperature. The threshold presents the defined value; the detected temperature is correct if the difference is smaller than the threshold; otherwise, the system will re-detect the temperature.

Regarding humidity, the study uses the evaporation equation presented by Priestley (1970) to judge whether the soil evaporation fulfills the prediction. Moreover, we combine weather forecast data to check whether it will rain. The evaporation equation is *ET*_0_ = α*S*(*Rn* − *G*)/(*s* + γ), where *Rn* is the net radiation, α is the equilibrium evaporation parameter, *G* is the sensible heat flux, and γ is the humidity. A large evaporation value means that the soil humidity is low, and the condition is normal. The study uses weather forecast information to judge whether the sensor is detecting correctly and further improve the correctness of LSTM.

### 3.4. Long Short-Term Memory Prediction

The study conducts Multivariate LSTM to manage the watering and detect the farm conditions. Firstly, the experiment judges whether the targeted crop can grow under the weather conditions as forecasted; if not (for example, when the temperature is too low or too high or when it will rain heavily), the system will send an alarm to notify the farmer. The equation is shown below:

**Algorithm 2:** Farm Environmental Detection algorithm.

//If the temperature is suitable for growing the targeted crop.
**if** (*ATe* < *PTe*_*h*_ and *ATe* > *PTe*_*l*_) **then**
Normal
**if** (QPF < *Threshold*) **then**
Normal
**else**
Alarm
**end if**
**end if**

The aforementioned equation checks whether the growing temperature (*ATe*) is set between the predicted highest (*PTe*_*h*_) and lowest temperatures (*PTe*_*l*_); if not, the system will notify the farmer to take care of the issue. On the other hand, the system will also confirm whether the Quantitative Precipitation Forecast (QDF) is higher than the threshold; if so, this means that the rain will be too heavy and will damage the crops.

**Figure 3:**
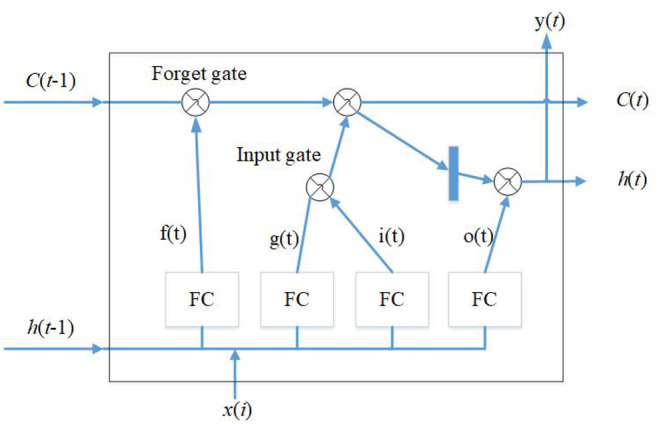
LSTM diagram.

The study utilizes soil humidity, temperature, the weather forecasted temperature, UV index, and QDF, and the calculated evaporation to make a watering prediction. Firstly, the evaporation equation, *ET*_0_ = α*S*(*Rn* − *G*)/(*s* + γ), is used to calculate the evaporation of the soil. The second step is to calculate the temperature curve. Because time and temperature are proportional, assuming that 12:00 to 14:00 is the period of the highest temperature, the percentage for that period is set as 100%; as the temperature decreases from 14:00 to 17:00, the percentages would be 70, 40, and 10%, and as the temperature increases between 07:00 and 12:00, the percentages would be 20, 40, 60, 80, and 100%. Therefore, the slopes of the current and predicted temperatures can be calculated from the equation *m* = Δ*y*/Δ*x*, where Δ*y* is the time percentage of the current temperature and Δ*x* is the predicted time percentage of the estimated temperature. After the calculation, using Multivariate LSTM for further prediction, the variable is calculated by the equation *x*_*i*_ = {*Hu*_*ct*_, *m, QDF, ET*_0_, *UV*}, where *Hu*_*ct*_ is the soil humidity and UV is the UV index. *x*_*i*_ is entered to conduct the LSTM analysis, and tanh is using to transfer the output value into a number between 1 and −1, as shown in Figure 3. According to the predicted time and the output value, the system will judge whether it is necessary to activate the sprinkler and environmental detection equipment. The system offered in this research can reduce electricity waste significantly and control water resources effectively to ensure a good growing environment for the crops. Building outdoor agricultural machinery based on LSTM enables farmers to set the desired watering system and environmental management parameters according to different crops, which can improve agricultural output and reduce agricultural labor use.

## 4. Performance

The performance of the system is described in sections Experiment Results of the System's Functions and LSTM Experimental Results.

### 4.1. Experiment Results of the System's Functions

**Figure 4:**
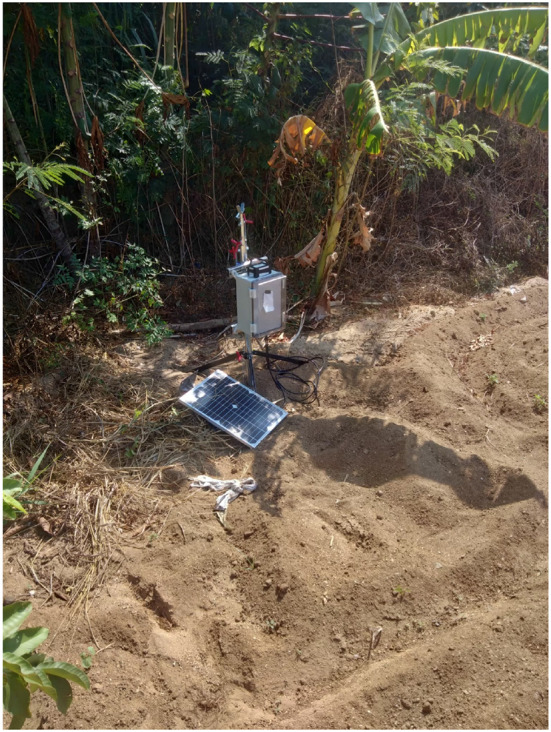
Experimental area.

**Figure 5:**
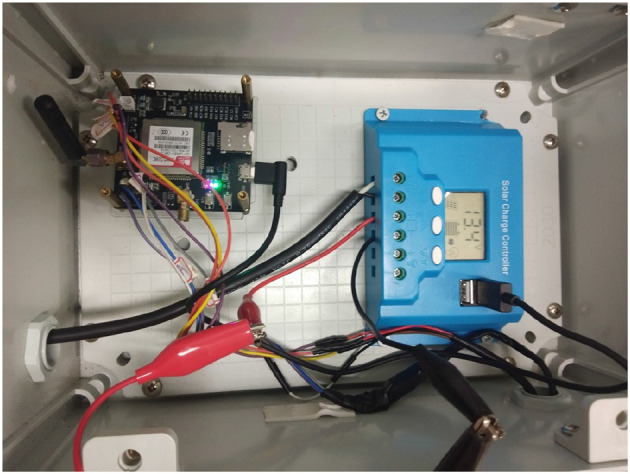
Experimental hardware.

The experimental area used in this research is an outdoor farm, as shown in Figure 4. The experiment uses a solar power system to store energy because there is no other source of electricity at the farm. The area is 0.2 hectares, and the equipment used is pictured in Figure 5; the machinery can be flexibly moved around the farmland, and the hardware and software equipped on the system are listed in Table 1. The machinery connects with the sensors and the development board through IoT; there are various types of sensors, such as barometric pressure and light sensors on the machinery, as well as a mini pumping motor, a 3-in-1 soil tester, and a solar power system. We set up a remote server that not only collects sensor data but also conducts LSTM analysis; the server further transfers the analytic results into readable signals for judging whether the system should activate the sprinkler system and initiate the environmental monitor.

**Table 1:** The software and hardware the proposed system.

**Hardware**	**Software**
IoT Development Board	XMPP Platform
Barometric Pressure Sensor	Python
Light Sensor	MySQL Server
Mini Pumping Motor	Windows 10
3-in-1 Soil Tester	
Solar Power System	

### 4.2. LSTM Experimental Results

**Figure 6:**
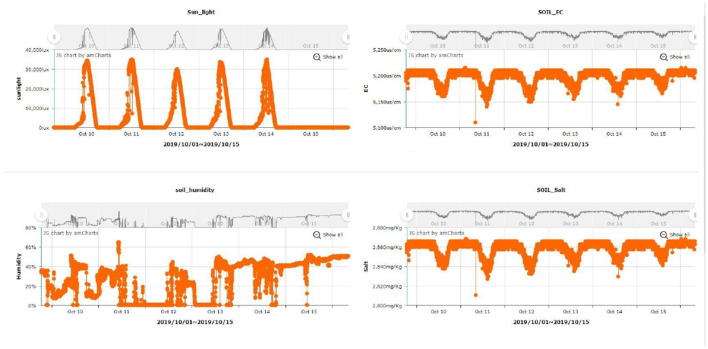
XMPP platform.

**Figure 7:**
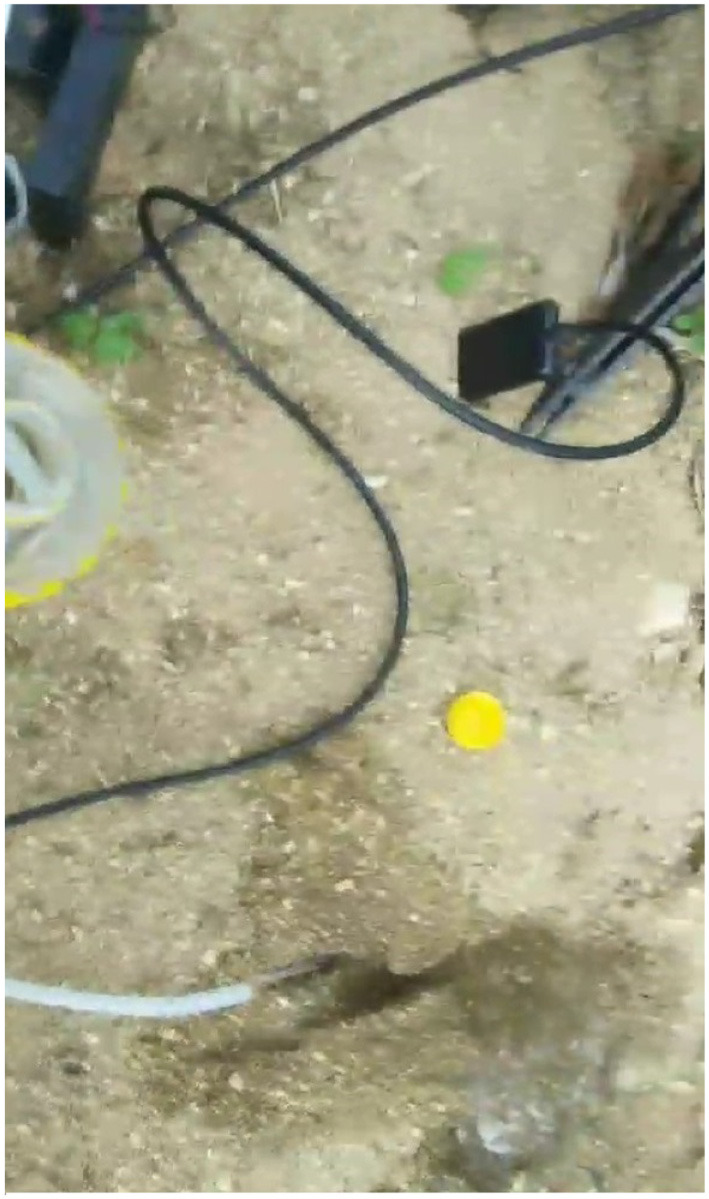
Activation of the sprinkler systems.

**Figure 8:**
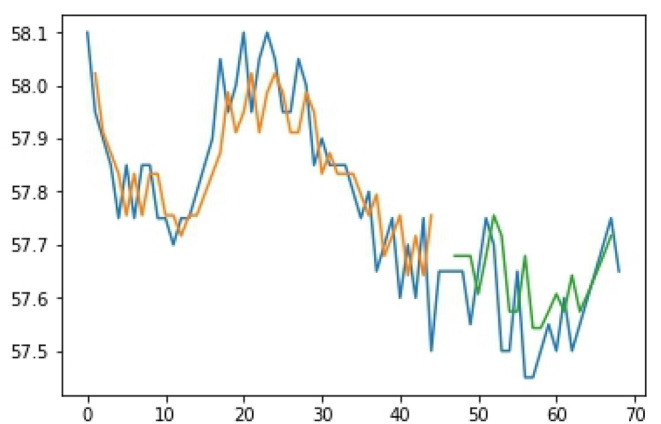
Prediction result of humidity.

**Figure 9:**
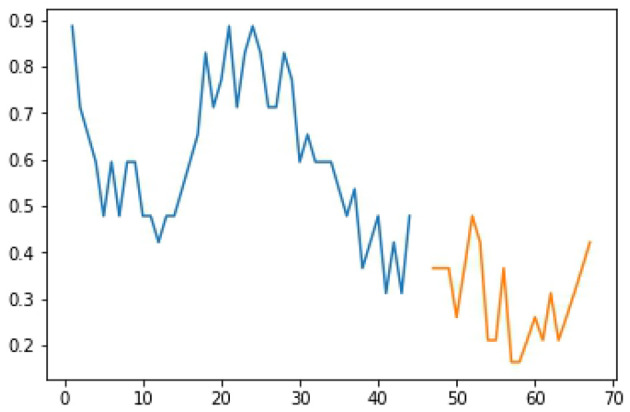
Prediction result of LSTM.

The study utilized LSTM to monitor the farm and select the timing for watering. Figure 6 shows the curves from the XMPP server and Figure 7 pictures the activation of the sprinkler system after the signals have been judged. The data from the Central Weather Bureau and the detected data from the environment were combined for a further prediction. The result is shown in Figure 8, where the blue line is the actual data, the orange line is the prediction result after training, and the green line is the result of the test data. In Figure 8, the number of samples is shown in the X-axis, while the Y-axis represents the soil humidity. The experimental results prove that the prediction approach offered in this study is extremely accurate. The LSTM prediction value is between 0 and 1, as Figure 9 shows; the blue line represents the training sample, the orange line shows the test sample, the X-axis is the number of samples, and the Y-axis is the prediction value. The threshold value is set to 0.4, and the sprinkler system will be activated when the value is lower than 0.4.

## 5. Conclusions

This study built outdoor agricultural machinery incorporating LSTM; the system can carry out watering automatically and monitor the farm conditions, fulfilling the purpose of an intelligent machine. The approach presented in this study can achieve the purpose of green energy effectively and reduce the waste of water resources. Through the implementation of LSTM, the system can analyze and predict the best timing for watering, which can avoid wastage of stored solar power and ensure an optimal growth environment for crops. Furthermore, the method can resolve the problem of an aging agricultural labor population and improve production output. The study successfully accomplishes the following functions. (1) The LSTM technique can predict the temperature and humidity of the soil precisely. (2) The suggested approach uses a solar power system and stores the electricity in a battery to reduce the workload of the power installation on the farm. (3) Through an IoT platform, the presented method is capable of predicting the best time to initiate the watering function and control water resources effectively. (4) The system presented in this study is mainly for outdoor application; the experimental results have proved that practical usage of the equipment is feasible. The experimental results show that the proposed approach was practical under testing; the LSTM experimental data demonstrated decent prediction performance. In the future, the authors aim to conduct a further study on monitoring multiple farms with a single server, which is expected to reduce the installation costs for farmers and enable the commercialization of the relevant equipment.

## Data Availability Statement

The datasets presented in this article are not readily available because the data also forms part of an ongoing study. Requests to access the datasets should be directed to hsinte@niu.edu.tw.

## Author Contributions

J-WZ proposed the experiment, compiled the data, and performed most of the analysis. C-HW and C-YL wrote most of the main text and contributed to the theoretical analysis and implications of the work. H-TW contributed to the analysis and discussion of the data. C-HW contributed to the theoretical analysis and implications of the work. All authors contributed ideas, discussed the results, and wrote the manuscript.

## Conflict of Interest

The authors declare that the research was conducted in the absence of any commercial or financial relationships that could be construed as a potential conflict of interest.
